# Prevalence and determinants of uterine rupture in Ethiopia: a systematic review and meta-analysis

**DOI:** 10.1038/s41598-020-74477-z

**Published:** 2020-10-19

**Authors:** Addisu Alehegn Alemu, Mezinew Sintayehu Bitew, Kelemu Abebe Gelaw, Liknaw Bewket Zeleke, Getachew Mullu Kassa

**Affiliations:** 1 College of Health Sciences, Debre Markos University, P.O.Box: 269, Debre Markos, Ethiopia; 2 College of Health Sciences, Wolita Sodo University, Wolita Sodo, Ethiopia

**Keywords:** Health care, Medical research, Risk factors

## Abstract

Uterine rupture is a serious public health concern that causes high maternal and perinatal morbidity and mortality in the developing world**.** Few of the studies conducted in Ethiopia show a high discrepancy in the prevalence of uterine rupture, which ranges between 1.6 and 16.7%. There also lacks a national study on this issue in Ethiopia. This systematic and meta-analysis, therefore, was conducted to assess the prevalence and determinants of uterine rupture in Ethiopia. We followed the Preferred Reporting Items for Systematic Reviews and Meta-Analyses (PRISMA) guidelines for systematic review and meta-analysis of studies. All observational published studies were retrieved using relevant search terms in Google scholar, African Journals Online, CINHAL, HINARI, Science Direct, Cochrane Library, EMBASE and PubMed (Medline) databases. Newcastle–Ottawa assessment checklist for observational studies was used for critical appraisal of the included articles. The meta-analysis was done with STATA version 14 software. The *I*^2^ test statistics were used to assess heterogeneity among included studies, and publication bias was assessed using Begg's and Egger's tests. Odds ratio (OR) with a 95% confidence interval (CI) was presented using forest plots. A total of twelve studies were included in this study. The pooled prevalence of uterine rupture was 3.98% (95% CI 3.02, 4.95). The highest (7.82%) and lowest (1.53%) prevalence were identified in Amhara and Southern Nations, Nationality and Peoples Region (SNNPR), respectively. Determinants of uterine rupture were urban residence (OR = 0.15 (95% CI 0.09, 0.23)), primipara (OR = 0.12 (95% CI 0.06, 0.27)), previous cesarean section (OR = 3.23 (95% CI 2.12, 4.92)), obstructed labor(OR = 12.21 (95% CI 6.01, 24.82)), and partograph utilization (OR = 0.12 (95% CI 0.09, 0.17)). Almost one in twenty-five mothers had uterine rupture in Ethiopia. Urban residence, primiparity, previous cesarean section, obstructed labor and partograph utilization were significantly associated with uterine rupture. Therefore, intervention programs should address the identified factors to reduce the prevalence of uterine rupture.

## Introduction

Uterine rupture is a tearing of the gravid uterine wall during pregnancy or delivery commonly on its lower part^1–3^. The tear can extend to the uterine serosa and may involve the bladder and broad ligament^4,5^. Disruption of uterine wall displaces the fetus into the abdomen, causes severe asphyxia and perinatal death and may necessitate massive transfusion or hysterectomy because of massive maternal bleeding^4,6^.

Uterine rupture is a rare 0.07%^7^ obstetric complication worldwide^8^ but it is a serious life-threatening which can adversely affect subsequent pregnancies^9^, and associated with significant and high maternal and fetal morbidity and mortality. It is one of the major public health concerns^2,10^ with 33% of maternal fatality rate and 52% of perinatal mortality rate^11,12^. The prevalence of uterine rupture in Ethiopia is higher (16.68%)^2,13,14^ compared to 1.3% in less developed countries^7^. Moreover, maternal morbidity and mortality are important public health issues in Ethiopia^15,16^.

Previous studies reported several factors that were associated with uterine rupture. The main reason for uterine rupture is the rapid increase in the number of previous cesarean deliveries. Additionally, factors like induction of labor, birth weight, gestational age and maternal characteristics were also associated with uterine rupture^11–13,17–19^. Women who had a previous cesarean section and whose labor was induced with uterotonic drugs also have an increased risk of uterine rupture and its subsequent complications^20–22^. Trial of labor after cesarean section (TOLAC) has comparable complication^23,24^ for most pregnancies with history of previous cesarean section^25^, although it has a high success rate^26^. However, recent reports showed that the rate of TOLAC is reducing^27^, despite the increasing rate of cesarean section globally^28^.

Identification of the factors associated with uterine rupture is one of the interventions to reduce the problem. Additionally, prompt diagnosis and timely identification of high-risk women, prompt diagnosis was also recommended^29^. Additionally, a more vigilant approach to prevent prolonged and obstructed labor, use of partograph^30^, quick referral to a well-equipped center and prevention of other obstetrics complications^14^ are key strategies to prevent uterine rupture.

Only few studies were conducted in Ethiopia on the prevalence of uterine rupture, although most of them focused on limited geographical areas. Therefore, we conducted this systematic review and meta-analysis to assess the pooled prevalence and determinants of uterine rupture in Ethiopia. The findings of the study will help to design effective strategies on the prevention strategies of uterine rupture in limited resource settings.

## Methods

### Search strategy and study selection

We conducted this systematic review and meta-analysis of all observational published studies to assess the pooled prevalence and determinants of uterine rupture in Ethiopia. Retrieving of the included studies was done in different databases such as Google scholar, African Journals Online, CINHAL, HINARI, Science Direct, Cochrane Library, EMBASE and PubMed (Medline) without restricting the study period. The Preferred Reporting Items for Systematic Reviews and Meta-analysis (PRISMA) guideline was strictly followed during systematic review and meta-analysis^31^.

A combination of search terms that best describe the study variables were used to retrieve articles. These include risk factors, determinants, predictors, factors, magnitude, prevalence, incidence, uterine rupture, laparotomy, hysterectomy, and Ethiopia. The terms were combined using "OR' and "AND" Boolean operators. Additionally, reference list of the already identified articles were checked to find additional eligible articles but were missed during the initial searching.

### Inclusion criteria

*Study design* All observational studies were included.

*Study period* Studies conducted until August 2018 were included.

*Participants* Women who had given birth at least once before data collection period of the included studies.

*Language* Only articles written in English language were included.

*Publication status* All studies regardless of publication status were considered.

### Exclusion criteria

Studies which we couldn’t access texts after three emails to the cross ponding authors were excluded.

### Outcome measure

Prevalence uterine rupture was the main outcome of this systematic review and meta-analysis. The pooled prevalence of uterine rupture was determined considering studies in which the status of uterus after delivery was reported. Additionally, determinants of uterine rupture among mothers were the outcome of this study.

### Data extraction

Data for this study were extracted from the included articles using data extraction checklist. Data extraction was made using Microsoft Excel sheet. Two of the authors (AAA and LBZ) participated in extracting data from the included studies. The data extraction checklist contains variables like author name, publication year, study design, sample size, and exposure characteristics that included the prevalence, partograph utilization, augmentation, residence, obstructed labor, previous Caesarean section (C/S) and antenatal care visit (ANC).

### Quality assessment

An intensive assessment of all articles included in this study was done by the two authors (AAA, MSB, KAG and LBZ). Newcastle–Ottawa assessment checklist^32^ for observational studies was used to assess the quality of each study included in this research. The tool has three sections. The first section was on methodological assessment and rated out of five stars, and the second section was on comparability evaluation and was rated out of three stars. The third section of the quality assessment tool was on assessing statistical analysis and outcome for each included study. There was a joint discussion between the authors for uncertainty, and the mean quality score was used to decide the quality of the included studies in the meta-analysis. Finally, studies scored ≥ 6 were grouped as having high quality.

### Statistical analysis, risk of bias and heterogeneity

Important data extracted from each primary (original) study through Microsoft Excel were exported to STATA version 14 software for analysis. Then, standard for each included studies was computed using Binomial distribution formula. To determine the pooled estimate metan STATA command was computed considering random-effect model. Forest plots with 95% confidence interval (CI) were used to present the findings of the study. The weight of each study is described by the size of each box, whereas the crossed line shows the CI at 95%. Publication bias was also assessed using Egger’s and Begg’s tests, and a *p*-value of less than 0.05 was used to declare its statistical significance^33,34^. Due to the presence of heterogeneity among^33^, subgroup analysis was computed considering the geographical region in which the studies were conducted.

## Results

### Selection of included studies

**Figure 1:**
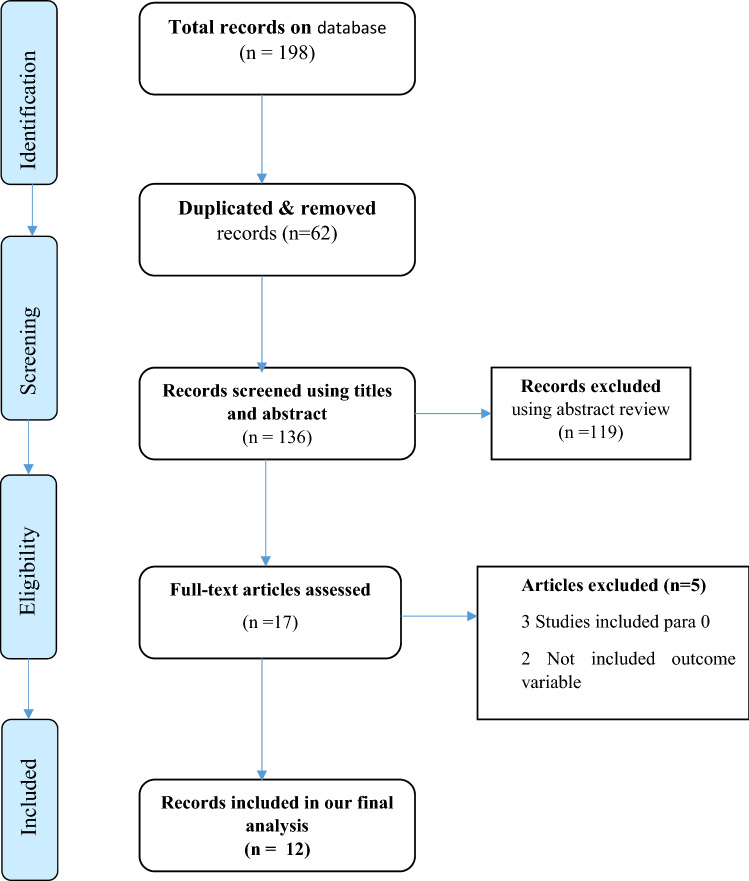
PRISMA diagram showing studies utilized for systematic and meta-analysis of uterine rupture in Ethiopia.

Database search resulted in a total of 198 research articles. Duplicated studies (n = 62) through their titles and abstracts were removed. Studies that passed abstract review were also screened using their title. Finally, a total of twelve studies were included in the current systematic review and meta-analysis (Fig. 1).

### Description of included studies

The characteristics of all included studies were presented in Table 1. Except one study, which used a retrospective cohort study design^34^, eight were cross-sectional studies^3,15,35–40^ and there were case–control studies^41–43^. This study included studies conducted from 1995 to 2018 on uterine rupture in Ethiopia. From this, four were conducted in Amhara region^3,35,37,44^, three were in Oromia region^36,38,40^, and two were conducted in SNNPR^34,39^. From the cross-sectional and retrospective studies, a total of 40,012 participants were included, observational studies and was used as the sample of in determining the pooled prevalence of uterine rupture. Additionally, 340 cases and 850 controls were included from case–control studies for the factor analysis, in addition to the sample used for prevalence estimation (Table 1).

**Table 1:** Descriptions of the studies utilized in the meta-analysis.

Study ID	Study design	Prevalence	Sample	Region	Quality
Admassu et al.^35^	Cross-sectional	3.8	1830	Amhara	9
Chamiso et al.^36^	Cross-sectional	2.6	2185	Oromia	9
Akine Eshete^34^	Retrospective cohort	1.8	2498	SNNPR	8
Getahun^14^	Cross-sectional	16.68	750	Amhara	8
Aliyu^3^	Cross-sectional	9.5	854	Amhara	6
Astatikie et al.^15^	Cross-sectional	2.44	10,379	Amhara	9
Woldeyes et al.^38^	Cross-sectional	1.6	2737	Oromia	7
Alemayehu^40^	Cross-sectional	3.7	10,270	Oromia	6
Mengistie^39^	Cross-sectional	1.6	8509	SNNPR	7

Study ID	Case–control design				
	Cases	Controls	Region	Quality	
Abebe et al.^45^	144	288	Oromia	8	
Marie et al.^41^	112	224	Tigray	7	
Solomon et al.^43^	84	338	Tigray	7	

### Prevalence of uterine rupture in Ethiopia

**Figure 2:**
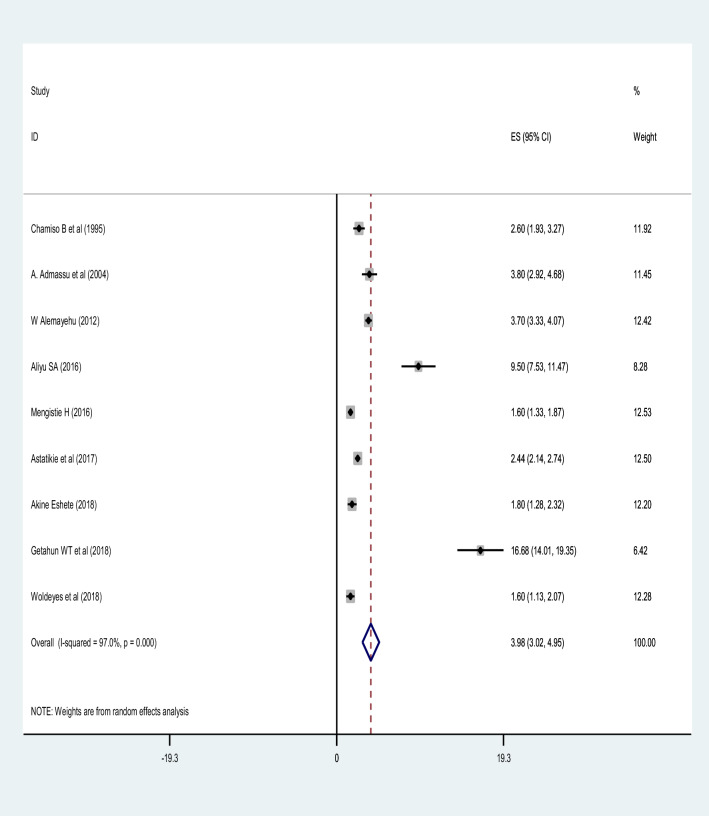
A forest plot describing the pooled prevalence of uterine rupture in Ethiopia.

**Figure 3:**
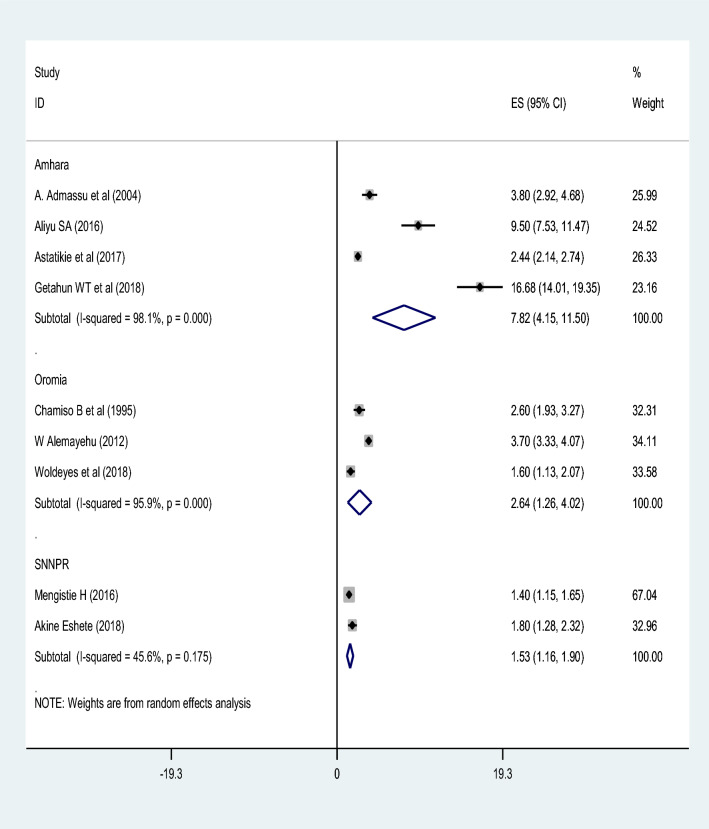
A forest plot shows the subgroup prevalence analysis of uterine rupture by study region.

The prevalence of uterine rupture using the included studies ranged from 1.4 to 16.68%^38,40^. The pooled prevalence of uterine rupture in Ethiopia was 3.98% (95% CI 3.02, 4.95). The random-effect model was used to analyze the pooled prevalence, however, a high and significant heterogeneity among the included studies (*I*^2^ = 97.3%; P-value ≤ 0.001) was observed (Fig. 2). Based on the subgroup analysis by study region, the highest prevalence of uterine rupture was in Amhara region 7.82% (95% CI 4.15, 11.50) and the lowest was in SNNPR 1.53% (95% CI 1.16, 1.90). However, there was significant heterogeneity in the included studies. Trim and fill meta-analysis was also conducted (Fig. 3).

### Factors associated with uterine rupture

The current review identified different factors associated with uterine rupture in Ethiopia. Significantly associated factors were residence, parity, history of cesarean section, obstructed labor and partograph utilization.

### Maternal residence

**Figure 4:**
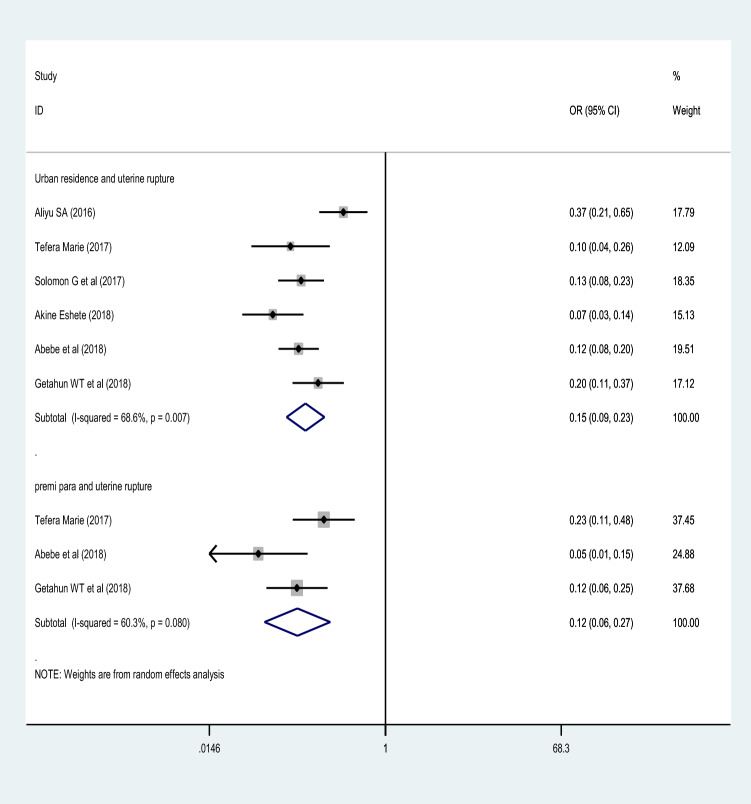
A forest plot describing the association of residence and parity with uterine rupture.

The maternal residence was significantly associated with uterine rupture. Using the studies included in group of meta-analysis^3,35,38,42–44^, women who live in urban areas were 85% less likely to have uterine rupture (OR = 0.15 (95% CI 0.09, 0.23) compared to women living in rural areas. Random effect model of analysis was used. The heterogeneity test showed statistically significant heterogeneity; I^2^ = 68.6%, p-value = 0.007. However, there was no significant publication bias (Begg’s and Egger’s test for, and P-value = 0.598 and 0.851, respectively) (Fig. 4).

### Parity

This group of analysis was conducted using three studies^38,42,43^. The meta-analysis finding showed parity as a strong predictor of uterine rupture. Women who were parity one were 88% less likely to have uterine rupture (OR = 0.12 (95% CI 0.06, 0.27)). There was no statistically significant heterogeneity among the included studies (*I*^2^ = 60.3%, *p-*value = 0.090) and no publication bias with Egger's and Begg's test of *P-*value = 0.964 and 0.602, respectively (Fig. 4).

### Previous cesarean section

**Figure 5:**
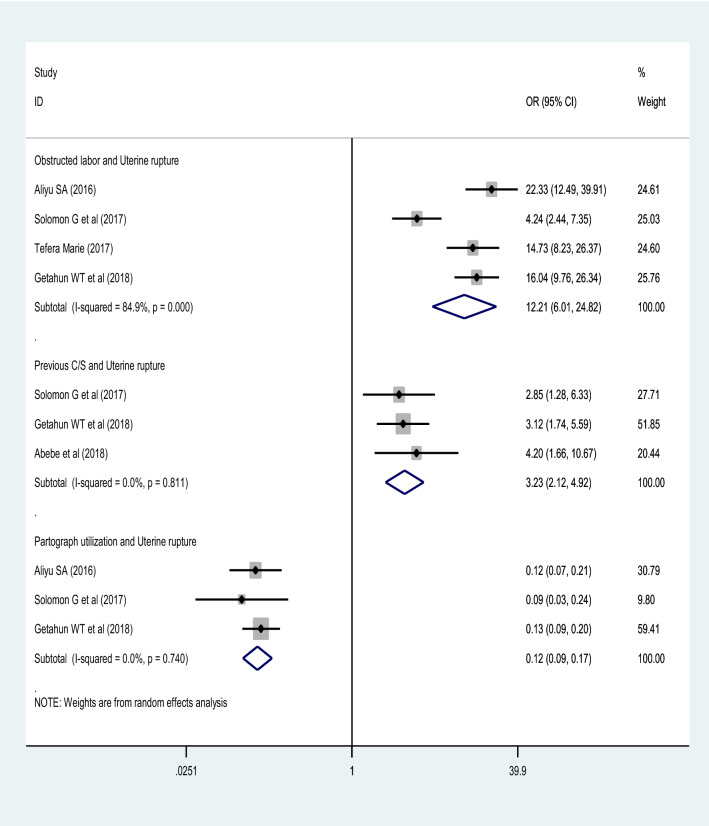
A forest plot describing the associations of obstructed labor, previous C/S, and partograph utilization with uterine rupture.

A strong association was observed between previous cesarean section with uterine rupture. Women who had previous cesarean section were 3.23 times more likely to develop uterine rupture compared to those who had no such history (OR = 3.23 (95% CI 2.12, 4.92)). This was true for all studies included in this analysis^38,43,44^. Using the random effect model of analysis and the *I*^2^ statistics (0.0%), there was no significant heterogeneity (Fig. 5).

### Obstructed labor

This systematic and meta-analysis included four studies^3,38,42,44^ to check the effect of obstructed labor on uterine rupture, and a significant association was observed. Women who were diagnosed for obstructed labor were more than twelve times more likely to have uterine rupture (OR = 12.21 (95% CI 6.01, 24.82)). The analysis was conducted using the random-effects model. The *I*^2^ statistics (84.9%) showed high heterogeneity, but Egger’s test showed no evidence of publication bias (*p*-value = 0.962) (Fig. 5).

### Utilization of partograph

A significant association was also observed between partograph utilization and uterine rupture using data of three studies^3,38,44^. After a random effect model analysis, mothers whose labor were attended using partograph were 88% less likely to have uterine rupture (OR = 0.12 (95% CI 0.09, 0.17)). *I*^2^ test statistics (0.0%) showed no heterogeneity. Both Egger’s (*p-*value = 0.117) and Begg’s (*p*-value = 0.118) tests also showed no publication bias (Fig. 5).

## Discussion

Reducing rates of primary cesarean section helps to reduce complications related to uterine rupture^2^. There is also an improvement in uterine rupture reduction with the implementation of nationally adopted guidelines on TOLAC^46^. Quality obstetric care, antenatal and family planning services with complete packages are important interventions in the reduction uterine rupture^1,10,43^.

In this study, we have estimated the national level of uterine rupture in Ethiopia. Our findings showed that the pooled prevalence of uterine rupture in Ethiopia was 3.98% with higher variability among regional states of the country, 1.53% in SNNPR to 7.82% in Amhara region. This estimated pooled prevalence of uterine rupture in Ethiopia is higher than nationwide studies conducted in western countries, 3.6 per 10,000 deliveries in Belgium^46^, 5.9 per 10,000 pregnancies in the Netherlands^47^, and 1.9 per 10,000 deliveries in United kingdom^13^. This much discrepancy and less prevalence of uterine rupture in developed countries^1^ might be due appropriate application of TOLAC to reduce repeated cesarean sections in developed countries^4^. Additionally, it could also be due to the higher prevalence of home delivery^48^, less optimal ANC attendance^49^, and delay in seeking healthcare services^50,51^.

In agreement with previous studies done in Ethiopia^38,43^ and Uganda^52,53^, this systematic review and meta-analysis study identified mothers who were residing in rural areas were more likely to face uterine rupture. The odds of uterine rupture were 85% times lower among urban residents compared with rural residents in this study. This might be due to the higher percentage of home birth in rural residents^48^ and their healthcare-seeking behavior depends usually on when complications arise.

Our finding also quantified obstructed labor as a strong determinant of uterine rupture in Ethiopia. Women who were diagnosed with obstructed labor were 12.21 times more likely to develop uterine rupture. Similarly, it is supported by previous studies in the same country in; Debre Markos^37^, Dessie^53^, and Bahir Dar^54^ and other nationwide studies outside the country in Uganda^55^, India^29^, Sweden^14^, Senegal and Mali^56^, and Niger^57^. This finding was also in line with a systematic review and meta-analysis in both developing and developed countries conducted by WHO^58^. This might be due to the higher teenage pregnancies in the corresponding countries secondary to low education attainment^59^ like in Ethiopia^60^, Uganda^61^, Niger^62^, India^63^ and Mali^64^ in which teenagers usually have less developed pelvic canal^69–67^ and have low ANC utilizations^68,69^.

This study also revealed women who were para one were 88% less likely to have uterine rupture than women with multiparity. This finding is supported by different prior studies in different countries in Ethiopia^42^, Norway^8,69^, Senegal and Mali^56^, Uganda^51^, and Israel^70^. It might be attributed to the fact as increasing parity increases the elasticity and strength of the uterine muscle (Myometrium) decreases^38,71^.

Likewise, previous cesarean section has been identified as a strong determinant of uterine rupture, women who had previous cesarean sections were more than 3 times more likely to be affected by uterine rupture. This finding was similar to others studied in Ethiopia^42,53^, Norway^8^, Globally^58^, USA^71^, and Nigeria^72^. Recent findings show the increasing uterine rupture goes through increasing cesarean section ^29,73^ due to prior uterine scar since the cesarean section alters the elasticity properties of the myometrium and collagen birefringence^74^ makes the uterus easily ruptured.

Women for whom partograph was utilized were 88% less likely to have uterine rupture than women who had no partograph during childbirth. This finding is in agreement with previous studies done in the same country, Ethiopia^3,37,75^. The reason behind might be that partograph predicts the possible complications of labor and helps to have timely decisions and interventions^76^. Uterine rupture is usually preceded by changes in uterine contractions^77^ prevented through proper partograph utilization^78^.

## Limitations and strength of the study

There is no study on uterine rupture conducted in Ethiopia at national level before this systematic and meta-analysis. Therefore, it shows the problem at the country level. However, it has limitation that the included studies’ designs were cross sectional and case control. Because of this the temporal relationships of outcome variable with determinants cannot be established.

## Conclusion

The pooled prevalence of uterine rupture in Ethiopia was high. Residence, partograph utilization, obstructed labor, previous C/S and parity were determinants of uterine rupture. The Ethiopian ministry of health should focus on preventing or reducing uterine rupture through facilitating and supervising of proper partograph utilization Moreover, unnecessary cesarean deliveries should be avoided. Additionally, intervention programs should also focus on the identified factors.

## Data Availability

All data utilized in this study are available from the corresponding upon request.

## Abbreviations

ANC: Antenatal care
C/S: Cesarean section
TOLAC: Trial of labor after cesarean section
CI: Confidence interval
OR: Odds ratio
SNNPR: Southern Nations, Nationality and Peoples Region
WHO: World Health Organization
SE: Standard error
PRISMA: The Preferred Reporting Items for Systematic Reviews and Meta-analysis
